# Size‐Controlled Formation of Polymer Janus Discs

**DOI:** 10.1002/anie.202105235

**Published:** 2021-08-26

**Authors:** Xiaolian Qiang, Steffen Franzka, Giada Quintieri, Xuezhi Dai, Chin Ken Wong, André H. Gröschel

**Affiliations:** ^1^ Physical Chemistry University of Münster Corrensstraße 28–30 48149 Münster Germany; ^2^ Center for Nanointegration Duisburg-Essen (CENIDE) and Interdisciplinary Center for Analytics on the Nanoscale (ICAN) University of Duisburg-Essen Carl-Benz-Str. 199 47047 Duisburg Germany

**Keywords:** 3D confinement, block copolymers, Janus discs, self-assembly, SPG membrane emulsification

## Abstract

A straightforward method is presented for the preparation of nano‐ to micrometer‐sized Janus discs with controlled shape, size, and aspect ratio. The method relies on cross‐linkable ABC triblock terpolymers and involves first the preparation of prolate ellipsoidal microparticles by combining Shirasu porous glass (SPG) membrane emulsification with evaporation‐induced confinement assembly (EICA). By varying the pore diameter of the SPG membrane, we produce Janus discs with controlled size distributions centered around hundreds of nanometers to several microns. We further transferred the discs to water by mild sulfonation of PS to polystyrene sulfonic acid (PSS) and verified the Janus character by subsequent labelling with cationic nanoparticles. Finally, we show that the sulfonated Janus discs are amphiphilic and can be used as efficient colloidal stabilizers for oil‐in‐water (O/W) emulsions.

## Graphical abstract

Nano‐ to micrometer‐sized Janus discs were fabricated from ABC triblock terpolymers through cross‐linking and disassembly of prolate ellipsoidal particles. By varying the pore diameter of a Shirasu porous glass (SPG) membrane, the size of prolate ellipsoidal particles and the corresponding Janus discs can be well controlled.

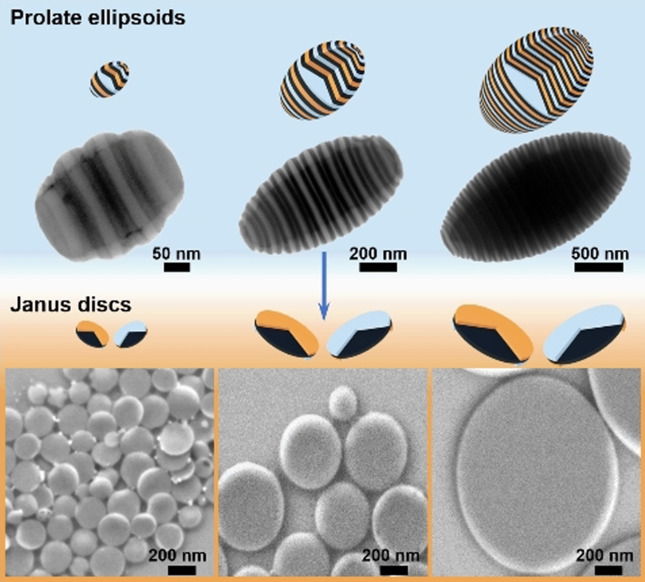

Janus particles (JPs) consist of two strictly separated sides with different physical properties.[^1^, ^2^, ^3^, ^4^] Since the first introduction of JPs based on polystyrene (PS)/poly(methyl methacrylate) (PMMA),^[5]^ JPs with controllable chemical asymmetry have been synthesized from diverse sources (organic, inorganic, hybrid, biological).^[6]^ Owing to advances in particle synthesis, the shape of JPs could be varied from, e.g., dumbbells,[^7^, ^8^] mushrooms,[^9^, ^10^] cylinders,[^11^, ^12^] discs/platelets,[^13^, ^14^, ^15^, ^16^] and cup‐/bowl‐shapes.[^17^, ^18^] Since the amphiphilicity of JPs is paired with their colloidal nature, they act as colloidal surfactants and are expected to be more effective stabilizers for emulsions as compared to molecular surfactants (Pickering effect).[^19^, ^20^] The ability of JPs to stabilize liquid‐liquid interfaces strongly depends on particle shape.^[21]^ Theoretical and experimental studies have repeatedly shown that disc‐shaped JPs are superior at stabilizing emulsions due to their inability to rotate and detach from liquid‐liquid interfaces.[^21^, ^22^, ^23^, ^24^, ^25^, ^26^]

Most Janus discs reported to date are made of inorganic components through silicon etching,[^27^, ^28^] crushing silica capsules with different modification on in‐ and outside[^29^, ^30^] or selective grafting of polymer chains onto inorganic Janus discs.[^31^, ^32^, ^33^] As an alternative to inorganic Janus discs, polymeric Janus discs are especially attractive since their physical/chemical properties can be tailored in a broad range through monomer chemistry including the ability to respond to stimuli such as pH, temperature, light, solvent, etc.[^34^, ^35^] So far, however, comparatively little work has been done on polymer‐based Janus discs due to a lack of synthetic concepts.[^15^, ^35^] One existing strategy utilizes lamella‐lamella (*ll*) bulk morphologies of ABC triblock terpolymers that after cross‐linking can be mechanically fractured by sonication into Janus nanosheets.^[15]^ In many cases, these Janus discs are polydisperse, and exhibit fringes or a non‐circular contour. Recently, evaporation‐induced confinement assembly (EICA) of block copolymers (BCPs) has emerged as a powerful approach to prepare microparticles with well‐defined inner structure as well as JPs thereof through cross‐linking and redispersion.[^36^, ^37^] Polymer Janus discs were also obtained using equal‐sized polystyrene‐*b*‐poly(4‐vinylpyridine),^[13]^ but the process heavily relied on specific block chemistries. The above strategy also typically relies on conventional emulsification methods (e.g., vortex mixing/sonication), which generate polydisperse emulsion droplets and consequently polydisperse Janus discs.

Considering how particle size is known to influence the orientation and packing geometry of particles at liquid‐liquid interfaces,[^21^, ^38^, ^39^] it would be desirable to establish a straightforward method to synthesize polymer Janus discs with size control, and to study how their geometric properties affect their ability to stabilize liquid‐liquid interfaces.

Herein, we present a facile approach towards size‐controlled polymer Janus discs by combining Shirasu porous glass (SPG) membrane emulsification with EICA to yield uniform prolate ellipsoidal microparticles with axially stacked lamella‐lamella morphology. Here, the utilization of SPG membrane not only allows to generate uniform BCP microparticles, but more importantly, to control microparticle size using membranes with defined pore diameter. We use a triblock terpolymer, polystyrene‐*block*‐polybutadiene‐*block*‐poly(methyl methacrylate) (PS‐*b*‐PB‐*b*‐PMMA or S_32_B_40_M_28_^202K^; subscripts denote the weight fraction of respective blocks, superscripts denote the overall molecular weight in kg mol^−1^, Figure S1 for detailed characterization) that forms lamella‐lamella morphology in bulk (Figure S2).^[14]^ The middle PB block solely serves as a cross‐linkable block and in principle allows to control thickness (with overall *M*_n_) and disc asymmetry depending on the block composition (in a relatively broad range from *f*_PB_=25–50 wt %). As summarized in Scheme S1, the entire fabrication process includes three steps: a) SPG membrane emulsification to yield uniform oil‐in‐water (O/W) emulsion droplets, and in effect, uniform SBM microparticles after solvent evaporation, b) cross‐linking of the SBM microparticles and c) redispersion of Janus discs. Importantly, the size of the Janus disc can be tuned simply by changing the membrane pore diameter, here from *d*_pore_=0.3, 0.8, and 2.0 μm.

We first prepared uniform O/W emulsion droplets by passing a solution of SBM in chloroform (CHCl_3_, 10 g L^−1^) with nitrogen pressure through a SPG membrane into an aqueous surfactant solution containing sodium dodecyl sulfate (SDS, 5 g L^−1^). In line with a previous report,^[40]^ the operation pressure (*P*) which is ≈1.3–2.3 times larger than the critical pressure (*P*_c_) was crucial to generate droplets of sufficiently narrow size distribution (see Table S1 for further details). Depending on the membrane pore diameter (*d*_pore_=0.3, 0.8, and 2.0 μm), we were able to control the size of the O/W droplets, and in effect, the size of the resulting polymer particles. After emulsification, the oil phase (CHCl_3_) was evaporated under ambient conditions to yield solid SBM particles.

The resulting SBM particles were characterized by scanning electron microscopy (SEM) and transmission electron microscopy (TEM). From the SEM overview images (Figure 1 a–c), all SBM particles‐regardless of membrane pore diameter—adopt a prolate ellipsoidal shape as evidenced by their average aspect ratios (AR, mean length to diameter ratio *L*/*D*, Table 1) of 1.41, 1.73, and 1.82 for *d*_pore_=0.3, 0.8, and 2.0 μm, respectively. The particle anisotropy likely originates from the unidirectional (axial) stacking of the lamellae. The increase in AR with increasing membrane pore diameter is consistent with previous reports based on AB diblock copolymers.^[41]^ More importantly, the SBM particles are uniform with a coefficient of variation (CV) value of ≈15 % (Table 1). Irrespective of membrane pore diameter, the shape and internal structure of all three sets of SBM particles are identical. The PS, PB and PMMA microphases alternate in form of an axially stacked lamellar structure due to their equal‐sized block compositions (S_32_B_40_M_28_^202K^) as well as the comparable affinity for PS (*δ*=18.5 MPa^1/2^) and PMMA (*δ*=19.0 MPa^1/2^) to the SDS/water interface.^[42]^ In TEM (Figure 1 d–f), the three microphases can be clearly identified: PS appears grey, PB dark due to osmium tetroxide (OsO_4_) staining, and PMMA bright; the PMMA lamella appears thinner in TEM due to its degradation under the *e*‐beam.^[43]^ Grey scale analysis (Figure S3) revealed that the lamella thickness (*T*=*L*_0_/2, Table 1) were 0.037 μm, 0.049 μm, and 0.052 μm for *d*_pore_=0.3 μm, 0.8 μm, and 2.0 μm, respectively. It is reasonable that the lamella thickness increased with larger membrane pore diameter. The SBM polymer chains are in spherical confinement within the emulsion droplets, where the confinement degree (ratio of *d*_pore_ to *L*_0_=0.0323 μm of SBM bulk film, Figure S2) became weaker with increasing membrane pore diameter (Table 1) and the effect of confinement is minimal when *d*_pore_/*L*_0_>40 (*d*_pore_=2.0 μm in our case).[^36^, ^44^] Therefore, the polymer chains packed in a looser manner with larger membrane pore diameter, resulting in a thicker lamella thickness.

**Figure 1:**
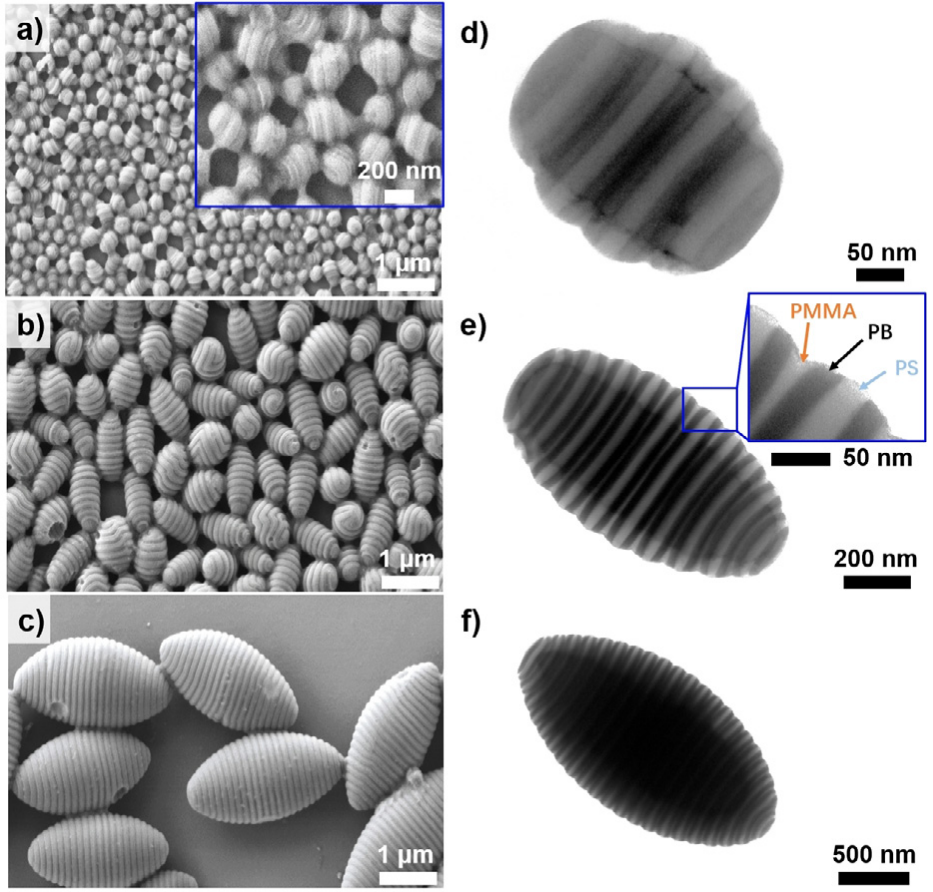
SBM prolate ellipsoidal particles generated from different membrane pore diameter. a–c) SEM overview and d–f) TEM images of SBM particles produced from different membrane pore diameter: a, d) *d*_pore_=0.3 μm, b, e) *d*_pore_=0.8 μm, and c, f) *d*_pore_=2.0 μm.

**Table 1:** Dimensions (length (*L*), diameter (*D*), aspect ratio (AR)), periodicity (*L*_0_), lamellar thickness (*T*) and confinement degree of SBM prolate ellipsoidal particles, and dimensions (diameter (*D*), height (*H*)), aspect ratio (*α*), number‐average area (*A*_n_), weight‐average area (*A*_w_), area dispersity (*A*_w_/*A*_n_) of Janus discs prepared from different membrane pore diameter (*d*_pore_).

*d*_pore_	Prolate ellipsoidal particles						Janus discs					
[μm]	*L* [μm]	*D* [μm]	AR	*L*_0_ [μm]	*T* [μm]	confinement degree	*D* [μm]	*H* [μm]	*α*	*A*_n_ [μm^2^]	*A*_w_ [μm^2^]	*A*_w_/*A*_n_
0.3	0.33±0.06	0.24±0.03	1.41	0.073	0.037	9	0.19±0.05	0.055	3.45	0.030	0.035	1.17
0.8	0.89±0.11	0.52±0.07	1.73	0.097	0.049	25	0.41±0.11	0.056	7.19	0.144	0.177	1.23
2.0	2.40±0.27	1.33±0.14	1.82	0.103	0.052	62	1.13±0.30	0.059	18.83	1.139	1.365	1.20

To produce Janus discs, we first cross‐linked the double bonds in the PB phase using OsO_4_. Subsequent redispersion in tetrahydrofuran (THF, good solvent for all three blocks) led to Janus discs with PS on one side and PMMA on the other side, and a central sandwiched PB phase. Figures 2 a–c show SEM images of the Janus discs obtained from SBM particles with *d*_pore_=0.3, 0.8, and 2.0 μm (see also Figure S4 for TEM images). It is obvious that the disc contour is strictly circular for all disc sizes. As summarized in Figure 2 d, discs obtained from *d*_pore_=0.3 μm have an average diameter of 0.19±0.05 μm (blue bars), which increases to 0.41±0.11 μm (red bars) for *d*_pore_=0.8 μm, and finally reaches 1.13±0.30 μm (green bars) for *d*_pore_=2.0 μm. The corresponding area dispersity (*A*_w_/*A*_n_, where *A*_w_ is the weight‐average area and *A*_n_ is the number‐average area^[45]^) was determined to 1.17 for *d*_pore_=0.3 μm, 1.23 for *d*_pore_=0.8 μm, and 1.20 for *d*_pore_=2.0 μm, which corroborates the relative low area polydispersity of all presented disc sizes. For each pore diameter there is a natural size distribution that originates from the ellipsoidal shape of the microparticles, where the disc diameter gradually decreases from the equator towards the poles. Nevertheless, the size dispersity is greatly improved using the SPG method and ABC triblock terpolymers as evident from the frequency distribution.

**Figure 2:**
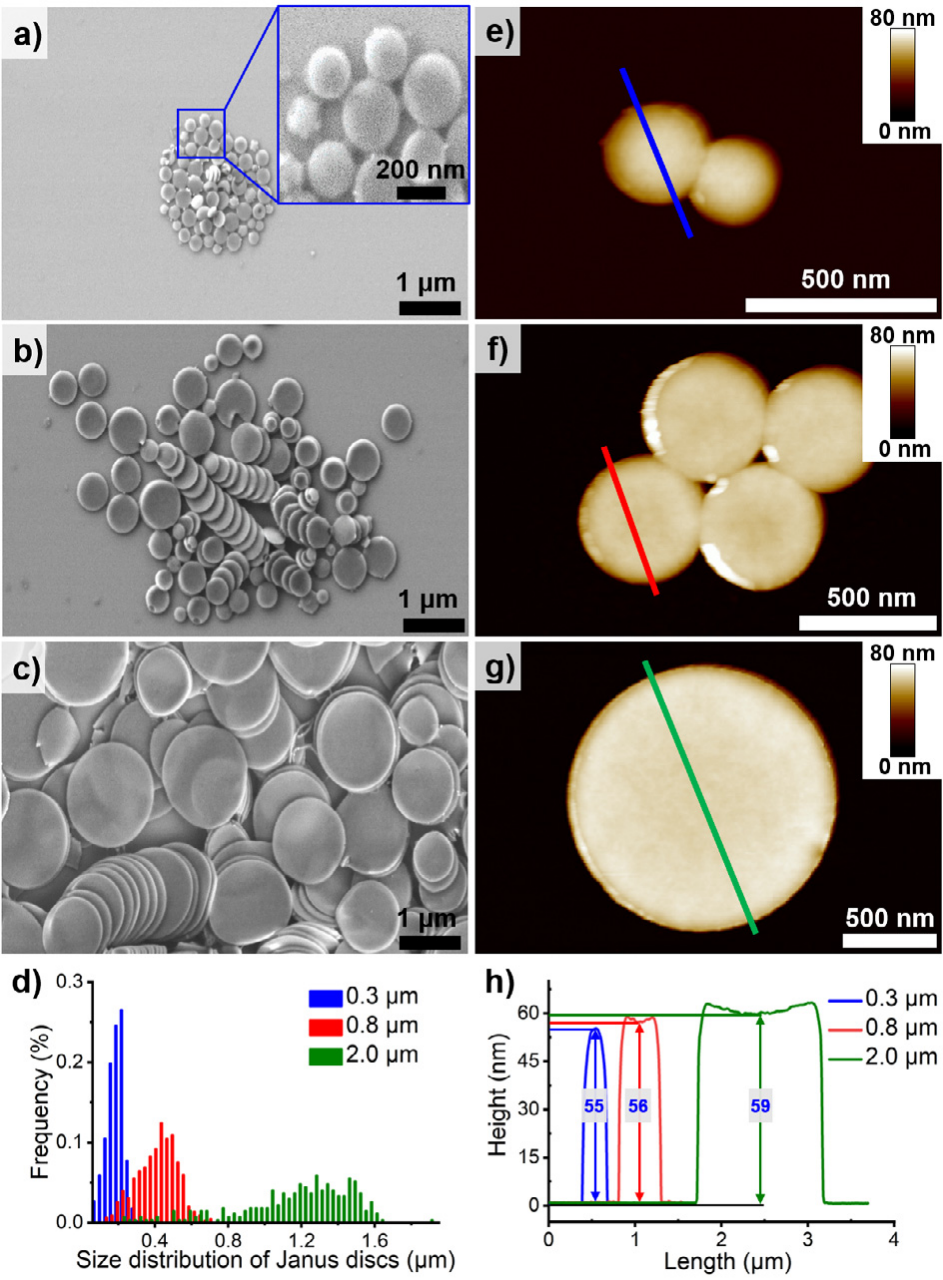
Janus discs after crosslinking of SBM microparticles. a–c) SEM images of discs for *d*_pore_=0.3 μm (a inset with magnification), *d*_pore_=0.8 μm (b), and *d*_pore_=2.0 μm (c). d) Size distributions for each disc sample based on more than 500 discs (measured by ImageJ). e–g) AFM images of discs for *d*_pore_=0.3 μm (e), *d*_pore_=0.8 μm (f), and *d*_pore_=2.0 μm (g). h) Height profile of the coloured lines in (e–g).

Atomic force microscopy (AFM) was used to analyze the height of the discs which is a measure of the disc thickness. Representative AFM images are shown in Figure 2 e–g for *d*_pore_=0.3 μm, 0.8 μm, and 2.0 μm, whereas height measurements resulted in the range of ≈55 nm, 56 nm, and 59 nm (Figure 2 h). The measured heights (*H*) of the discs are slightly larger than the lamella thickness (*T*) within the microparticle (Table 1), especially for the smallest membrane pore diameter. We assume that the denser packing of polymer chain under stronger confinement, i.e., smaller membrane pore diameter, will release more obviously after disassembly as THF is a good solvent for both non‐cross‐linked PS and PMMA blocks, resulting in a more significant difference between discs thickness and lamella thickness. Despite the large differences of the particle sizes, the discs display only minor differences in height, which we attribute to the constant overall molecular weight of SBM. With almost constant thickness, the three discs show large differences in shape anisotropy given by their aspect ratio *α*, i.e., diameter/height ratio. The method is able to achieve an increase in *α* from 3.45 to 18.83, simply by changing the membrane pore diameter from *d*_pore_=0.3 μm to *d*_pore_=2.0 μm (Table 1).

As the discs prefer to orientate parallelly to interfaces, the individual PS, PB, and PMMA domains and therefore the Janus character of the discs cannot be distinguished from TEM (Figure S4), SEM or AFM (although PS and PMMA show light differences in AFM).^[46]^ Thus, we applied a post‐modification approach to convert PS to poly(styrene sulfonic acid) (PSS) to transfer the Janus discs to water, where PSS is negatively charged at pH>1 (Figure 3, SI for details).[^47^, ^48^] Note that, the discs used for sulfonation must be cross‐linked with sulfur monochloride (S_2_Cl_2_) as those cross‐linked with OsO_4_ did not withstand the sulfonation conditions (see SI for cross‐linking details). The TEM images in Figure 3 a and b show S_2_Cl_2_ cross‐linked Janus discs prepared from *d*_pore_=2.0 μm before and after sulfonation. Neither S_2_Cl_2_ cross‐linking nor sulfonation had an obvious negative effect on the disc structure. AFM analysis (Figure 3 c and d) revealed a similar disc thickness before and after sulfonation. Fourier transform infrared (FT‐IR) spectroscopy (Figure S5) further verified the sulfonation of PS to PSS, as the sulfonated discs exhibit characteristic vibrations at 1015 cm^−1^, 1124 cm^−1^, and 1147 cm^−1^ corresponding to S=O, S−O, and O=S=O.^[49]^ The broad signal at around 3386 cm^−1^ proved the existence of O−H bonds from the sulfonic acid groups (SO_2_OH) and probably water absorption to the hydrophilic PSS side.^[50]^ The colloidal stability of the sulfonated Janus discs in water was confirmed by a zeta potential of −54 mV. To confirm the Janus character, we synthesized positively charged cationic gold nanoparticles (AuNPs)^[51]^ (SI and Figure S6) and attached them to the negatively charged PSS side of the Janus discs. Figure 3 e shows an SEM image of two Janus discs (TEM image in Figure S7): one disc with PMMA facing upwards (left) and another with the PSS/AuNPs complex facing upwards (right). The single rolled‐up disc in Figure 3 f further confirmed the Janus character, where the disc exhibited a nanoparticle‐free PMMA side and another complexed PSS/AuNPs side.

**Figure 3:**
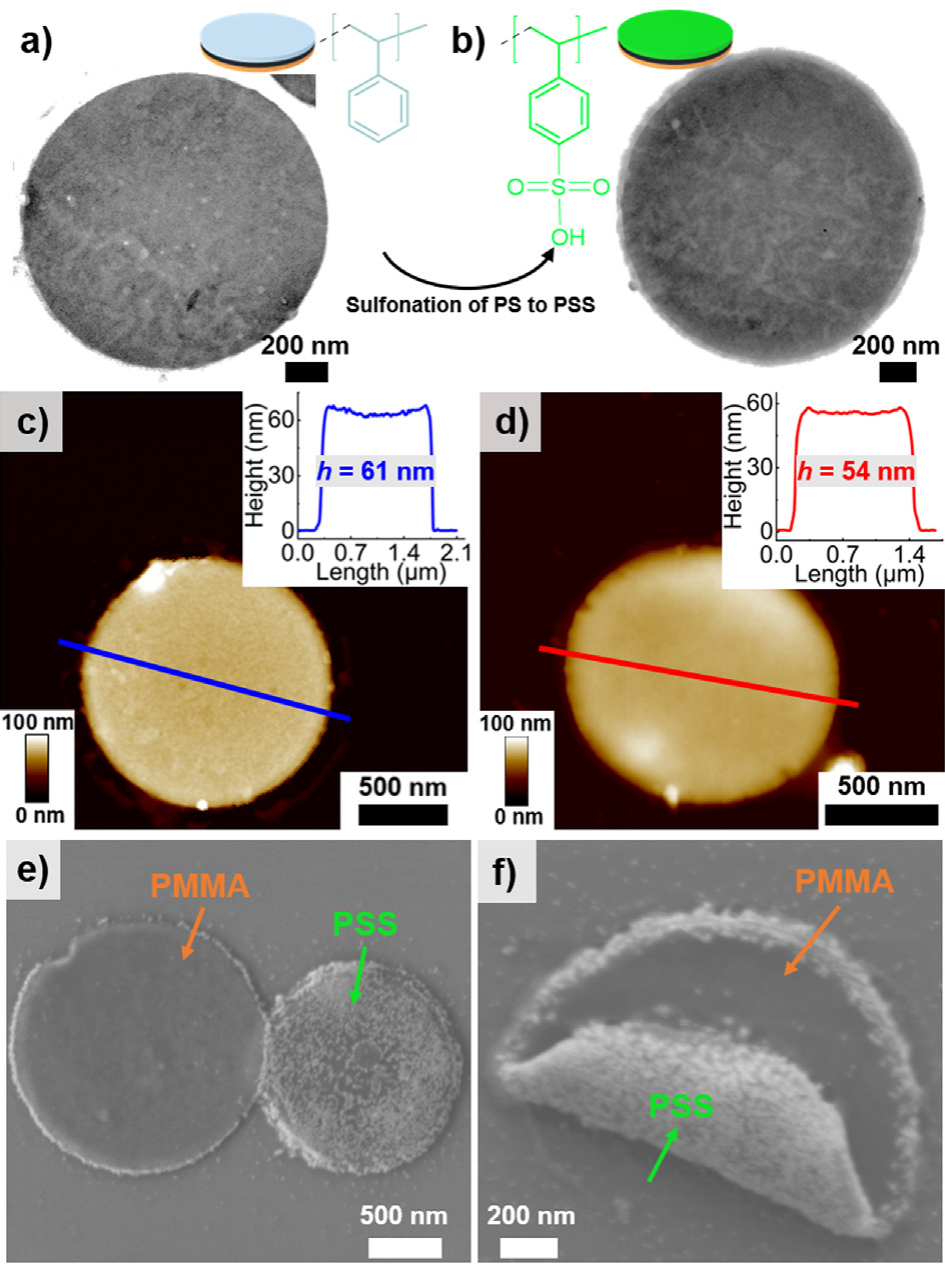
Sulfonation of PS to PSS from *d*_pore_=2.0 μm Janus discs. a, c) TEM and AFM images of S_2_Cl_2_ cross‐linked Janus disc (from THF). b, d) TEM and AFM images of sulfonated Janus disc (from water). Insets on the upper right corner in (c) and (d) are height profile measurements of the colored lines from (c) and (d), respectively. e) SEM image of sulfonated Janus discs incorporating with cationic AuNPs (from water). f) SEM image of single rolled up sulfonated Janus disc with AuNPs loaded on PSS side (from water).

Janus discs are known to effectively stabilize liquid‐liquid interfaces,[^21^, ^22^] however, the effect of Janus disc size on stabilization has not been investigated (as there was no means of controlling the size). We therefore sulfonated the three Janus disc sizes and studied how the disc diameter influences the stability of O/W emulsions (refer to SI for experimental details). As shown in Figure S8a, we found that a disc concentration of 0.05 wt % was sufficient to produce emulsions for all disc sizes. No noticeable difference was identified directly after emulsification. After leaving the emulsion to rest for 7 days (Figure S8b), the emulsion droplets accumulated at the toluene/water interface due to their higher density relative to toluene. Interestingly, the largest discs (*d*_pore_=2.0 μm) showed the largest volume of remaining stable emulsion droplets after 7 days (blue boxes in Figure S8b for comparison). This is reasonable since discs with larger surface area exhibit a higher energy barrier to rotate and detach from the oil/water interface.^[39]^ Janus discs have reportedly been used to stabilize Pickering emulsions. However, typical disc concentrations used are >0.30 wt %.[^13^, ^26^] In our case, a disc concentration of 0.05 wt % was sufficient to achieve long term stability of the emulsion. We decreased the disc concentration by 50‐fold to 0.001 wt % and emulsified a toluene/water mixture as before. Remarkably, we observed spherical toluene/water emulsion droplets (false‐colored red in Figure S8c) even at such a low disc concentration. This ultimately confirmed the extraordinary ability of our Janus discs in stabilizing O/W emulsions.

Overall, we have demonstrated a straightforward approach to produce uniform Janus discs with controllable size by synergizing the advantages of SPG membrane emulsification with EICA of ABC triblock terpolymers. The first step involves the fabrication of size‐controlled uniform prolate ellipsoidal SBM particles with stacked lamellae morphology using membrane pore diameter ranging from 0.3 μm to 2.0 μm. Subsequent disassembly of the ellipsoidal particles after cross‐linking and redispersion led to size‐controlled PS/PB/PMMA Janus discs. By sulfonating PS to negatively charged PSS, we show that cationic AuNPs can be absorbed exclusively on the sulfonated PSS face, thereby proving the discs’ Janus character. Finally, we showed that the PSS/PB/PMMA Janus discs are amphiphilic and can effectively stabilize O/W Pickering emulsions at an extremely low concentration (0.001 wt %). We further found that larger Janus discs are more useful for producing larger volumes of stable emulsion droplets.

## Conflict of interest

The authors declare no conflict of interest.

## Supporting information

As a service to our authors and readers, this journal provides supporting information supplied by the authors. Such materials are peer reviewed and may be re‐organized for online delivery, but are not copy‐edited or typeset. Technical support issues arising from supporting information (other than missing files) should be addressed to the authors.

Supporting Information
