# Supplementary material for: Size‐Controlled Formation of Polymer Janus Discs
Source: Angew Chem Int Ed Engl. 2021 Aug 26;60(40):21668–72. doi: 10.1002/anie.202105235 (PMC8518367; doi:10.1002/anie.202105235)
Supplement: Supplementary file 1 — Supporting Information [file ANIE-60-21668-s001.pdf]

Angewandte Chemie
Eine Zeitschrift der Gesellschaft Deutscher Chemiker

Supporting Information

Size-Controlled Formation of Polymer Janus Discs

Xiaolian Qiang, Steffen Franzka, Giada Quintieri, Xuezhi Dai, Chin Ken Wong,* and André H. Gröschel**

anie_202105235_sm_miscellaneous_information.pdf

Table of Contents

Experimental Procedures 2
Materials 2
Methods 2
Results and Discussion 4
Operation pressure for different membrane pore diameter 4
Formation of size-controlled polymer Janus discs 4
Characterization of the used SBM triblock terpolymer 5
Grey scale analysis of SBM bulk film 6
Grey scale analysis of SBM prolate ellipsoidal particles 6
TEM images of Janus discs from different membrane pore diameter 7
IR spectra of Janus discs before and after sulfonation 7
TEM images of AuNPs 8
TEM image of sulfonated Janus discs loaded with AuNPs 8
Emulsion stabilization of sulfonated Janus discs 9
Supporting References 10
Author Contributions 10

Experimental Procedures

Materials

Sodium dodecyl sulfate (SDS, > 99%, Sigma-Aldrich), osmium tetroxide (OsO_4, 4 wt% in H_2O, Science Services), acetic acid anhydride (≥ 99%, Carl Roth), sulfur monochloride (S_2Cl_2, 98%, Aldrich), sulfuric acid (H_2SO_4, ≥ 95%, Aldrich), gold(III) chloride trihydrate ($HAuCl_4 \cdot 3H_2O$, 99.9%, Sigma), sodium citrate dihydrate ($C_6H_5Na_3O_7 \cdot 2H_2O$, ≥ 99%, Sigma), (11-mercaptoundecyl)-*N*,*N*,*N*-trimethylammonium bromide (MUTAB, Aldrich), octadecylamine (ODA, ≥ 99.0%, Aldrich), hydrochloric acid (HCl, 37%, VWR), Nile red (for microscopy, Sigma) were used as received. All solvents were of analytical grade and used as received. The sequential anionic polymerization of polystyrene-*block*-polybutadiene-*block*-poly(methyl methacrylate) triblock terpolymer (PS-*b*-PB-*b*-PMMA or SBM) was described before elsewhere.[1]

Methods

Preparation of SBM prolate ellipsoidal particles through SPG membrane emulsification. The SBM triblock terpolymer ($S_{32}B_{40}M_{28}^{202K}$, subscript denotes the weight fraction of the respective block; superscripts denote the molecular weight in $kg \cdot mol^{-1}$) was dissolved in chloroform ($CHCl_3$) at a concentration of 10 $g \cdot L^{-1}$. Stock SDS aqueous solution was prepared at a concentration of 5 $g \cdot L^{-1}$. In a typical experiment, 1 mL of polymer solution was emulsified in 20 mL of aqueous SDS solution using a Shirasu porous glass (SPG) membrane device.[2] Uniform SBM emulsion droplets were generated by extruding the disperse phase through the SPG membrane with a positive flow of nitrogen (Table S1 for working pressures) while keep stirring solution at 250 rpm. Membranes with different pore diameter (0.3 µm, 0.8 µm, and 2.0 µm) were used to manipulate the droplet size. $CHCl_3$ was allowed to evaporate for 2 days at room temperature after emulsification. The final particle suspensions with a concentration of about 0.5 $g \cdot L^{-1}$ were purified by dialysis against ultrapure water to remove the surfactant, SDS.

Preparation of SBM Janus discs. The PB middle block of the SBM was cross-linked with OsO_4.[3] For this, the particle solution was mixed with aqueous OsO_4 solution (4 wt %) (molar ratio of PB:OsO_4 = 1:1) for 2 hours in a closed vial. Excess unreacted OsO_4 was removed by evaporation overnight, and the particles were washed with ultrapure water *via* repeated centrifugation/redispersion cycles (6000 rpm, 20 min, 3 times). The particles were finally dispersed in tetrahydrofuran (THF, c = 0.2 $g \cdot L^{-1}$) and stirred for 2 days to obtain Janus discs. For the Janus discs from d_{pore} = 2.0 µm, four centrifugation/redispersion cycles with THF (6000 rpm, 2 seconds) was applied to remove most of the smaller discs.

Sulfonation of PS to PSS in SBM Janus discs. The particles were first cross-linked with S_2Cl_2 prior to the sulfonation step. For cross-linking, the particle solution was freeze-dried overnight beforehand. Then, an uncapped 7 mL vial containing the freeze-dried particles and an uncapped 3 mL vial containing 0.1 mL of S_2Cl_2 were placed in a closed container overnight at room temperature. The next day, the lid of the closed container was removed to evaporate excess S_2Cl_2. Finally, the cross-linked particles were dissolved in THF (c = 0.2 $g \cdot L^{-1}$) and stirred for 2 days to obtain the S_2Cl_2 cross-linked SBM Janus discs. For sulfonation, 1 mg of S_2Cl_2 cross-linked SBM Janus disc was redispersed in 5 mL of dichloroethane (DCE) and deoxygenated by bubbling with argon for 15 min. The dispersion was heated to 50°C under a positive flow of argon, after which 2-3 mL of 2.5 M of freshly prepared acetyl sulfate solution was added slowly with a syringe. The 2.5 M acetyl sulfate solution was prepared *via* the sequential addition of sulfuric acid (1.4 mL, 0.025 mol) and acetic acid anhydride (2.4 mL, 0.025 mol) in DCE (6.2 mL) at 0°C.[4,5] The reaction was allowed to proceed for 24 h before being quenched by adding 5 mL of methanol (MeOH) to the reaction mixture. DCE was distilled off on a rotary evaporator. The sulfonated Janus discs were washed with ultrapure water *via* three centrifugation/redispersion cycles (4000 rpm, 10 min), and finally dispersed in ultrapure water at a concentration of 1.0 $g \cdot L^{-1}$.

Synthesis of cationic gold nanoparticles (AuNPs). Citrate-AuNPs were synthesized according to the method reported before.[6] In brief, 100 mL of 2.2 mM sodium citrate aqueous solution was heated to the boiling point under vigorously stirring. Afterwards, 0.67 mL of 25 mM $HAuCl_4$ was injected to the solution. The mixture was heated for another 20 min and the colour of the reaction changed to red during the reaction time. Then, the mixture was allowed to cool down to room temperature and AuNPs were collected. Cationic-AuNPs were synthesized according to the method reported by Ras *et al.*[7] In brief, 6 mL of toluene was added to 30 mL of citrate-AuNPs water solution. 5 mmol ODA was added to the above mixture. Afterwards, the mixture was shaken vigorously in order to transfer the AuNPs to toluene. The organic phase was separated and washed with ultrapure water extensively. 3 mL of ultrapure water and 300 mL of MUTAB (4 mM in ethanol) were added subsequently and the mixture was shaken to transfer the ODA-AuNPs to the aqueous phase. By acidifying the mixture through addition of HCl, the transfer was completed.

Coordination of AuNPs to sulfonated side of Janus discs *via* an electrostatic attraction. 70 µL of sulfonated Janus discs (1 $g \cdot L^{-1}$ in ultrapure water) were mixed with 60 µL of cationic AuNPs (9×10^{-9} $mol \cdot L^{-1}$). During mixing, the cationic AuNPs gradually adsorb onto the sulfonated PS face of the Janus discs *via* electrostatic interaction between the cationic AuNPs and negatively charged –SO_3^- groups.

Preparation of Pickering emulsions. 0.4 mL of toluene containing 0.01 wt% Nile red and 0.8 mL of aqueous suspension containing different size and concentration of sulfonated Janus discs were added to a glass vial. The emulsification was performed through vortex mixing for 1 min. All Pickering emulsions were more than 30 min at a standstill before imaging.

Gel permeation chromatography (GPC). GPC measurements were performed on a 1260 Infinity (Polymer Standard Service, Mainz) instrument equipped with 3 SDV columns (pore sizes 103, 105, and 106 Å), and a refractive index and multi UV/VIS (190-950 nm) detector. THF with HPLC grade was used as eluent. The samples were eluted at a 1.0 mL min^{-1} flow rate at 40°C. For calibration, a narrow molecular weight polystyrene (PS) standard kit (Polymer Standard Service, Mainz) and WinGPC UniChrom software were used together.

Nuclear magnetic resonance (1H NMR). The spectra were recorded on a Bruker NEO 400 MHz spectrometer with deuterated $CDCl_3$ as the solvent.

Transmission electron microscopy (TEM). TEM images were recorded on a JEOL JEM-1400 Plus TEM operating at an acceleration voltage of 120 kV. Samples for TEM were prepared by placing a drop of the particle dispersion on a carbon-coated copper grid. After 30 s, the solution was blotted with a filter paper and the grid dried for at least 12 h at room temperature. The SBM prolate ellipsoidal particles were selectively stained with aqueous OsO_4 vapor for at least 2 h prior to imaging. Images were processed with ImageJ software (version 1.52i). The length (*L*), diameter (*D*) of the prolate ellipsoids and the diameter (*D*) of the Janus discs were determined based on statistical analysis of more than 200 particles measured by ImageJ.

Scanning electron microscopy (SEM). SEM images were recorded on a cryo-field emission Zeiss Crossbeam 540 FIB-SEM equipped with in lens-, chamber-, and energy-selective detectors for 16 Bit image series acquisition with up to 40,000 × 50,000-pixel resolution. Samples for SEM measurements were prepared by putting one drop particle dispersion on a silicon wafer. After 30 s, the solution was blotted with a filter paper and the wafer was dried for at least 12 h. Afterwards, a layer of 10 nm platinum was sputtered onto the samples using a Quorum PP3010T-Cryo chamber with integrated Q150T-Es high-end sputter coater and Pt-Cd target.

Fourier Transform Infrared (FT-IR) Spectroscopy. The spectra were recorded on a Digilab 3100 FT-IR Excalibur Series spectrometer.

Atomic Force Microscopy (AFM). AFM images of the Janus discs were recorded in air using a Bruker Dimension Icon with NanoScope V controller in tapping mode with RTESPA cantilevers (nom. F_R = 300 kHz, k_C = 40 N·m^{-1}, Bruker). AFM samples of the Janus discs dispersed in THF (0.2 g·L^{-1}) were prepared by spin-coating a small drop of the dispersion on a freshly cleaned (ethanol p.a.) piece of silicon wafer (10 × 10 mm^2) with a home-build spin-coater at 3200 rpm for 5 s. AFM samples of the sulfonated Janus discs dispersed in ultrapure water (1.0 g·L^{-1}) were prepared by depositing a drop of the dispersion also on freshly cleaned (ethanol p.a.) pieces of silicon wafer (10 × 10 mm^2), air dried for 30 min and then subsequently dry-blowing using a stream of nitrogen. Images were processed with Bruker's NanoScopeAnalysis software (version 1.9) and/or Gwyddion software (version 2.48).[8]

Zeta potential. The zeta potentials of sulfonated Janus discs and cationic AuNPs were conducted on a Nano ZS Zetasizer (Malvern Instruments) operating at 25°C. Sample was measured in disposable DTS 1060 capillary cells (Malvern Instruments).

Fluorescence Microscopy. Samples were prepared by sandwiching 20 µL of Pickering emulsion between two glass slides and sealed with vacuum grease. Images were acquired on an Olympus BX53 microscopy using a 10X objective lens.

Results and Discussion

Operation pressure for different membrane pore diameter.

Table S1. Operation pressure for different membrane pore diameter.

Membrane pore diameter (μm)	Critical pressure (P_c, MPa)	Operation pressure (P, MPa)	P/P_c
0.3	0.060	0.123	2.05
0.8	0.023	0.031	1.35
2.0	0.009	0.013	1.44

Critical pressure (P_c) is the minimal pressure for membrane emulsification, and can be calculated from the following equation:[9]

$$P_c = \frac{4\gamma \cdot \cos\theta}{d_{pore}}$$

where d_{pore} is the membrane pore diameter, γ is the interfacial tension between oil phase and water phase, and θ is the contact angle between the oil phase and the membrane surface. The interfacial tensions γ at the (PS-toluene)/(water-SDS) and (PMMA-toluene)/(water-SDS) interface were estimated according to previous reports.[10] We assume that γ for (PB-toluene)/(water-SDS) was the same as (PS-toluene)/(water-SDS); γ was independent of the BCP concentration; cosθ = 1.[2,11,12]

Formation of size-controlled polymer Janus discs

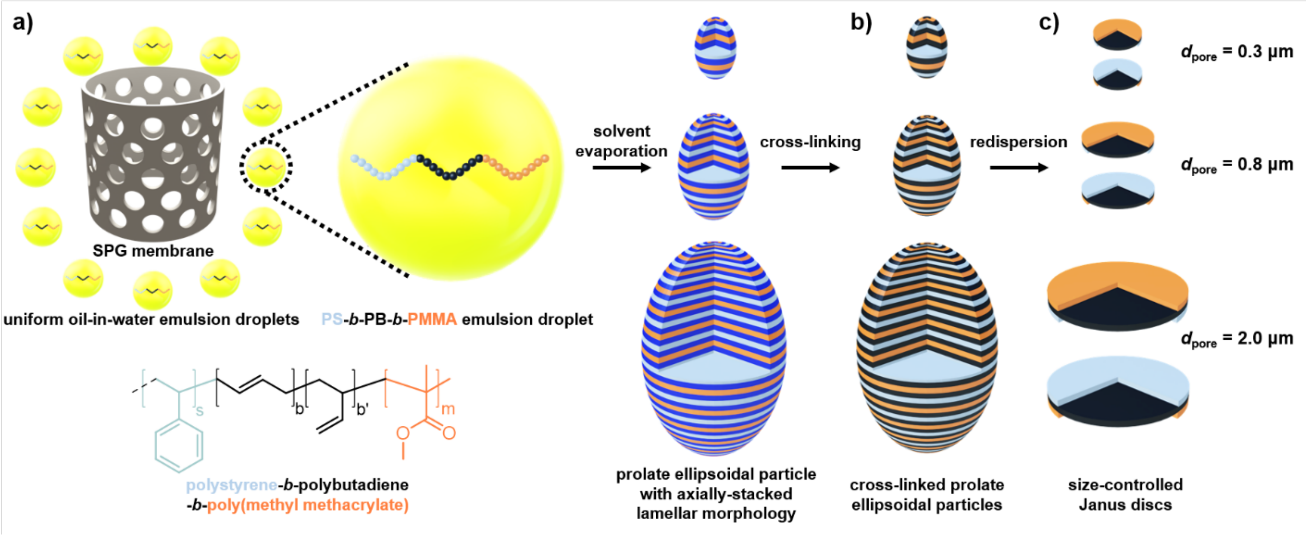

Scheme S1. Formation of size-controlled polymer Janus discs by synergizing SPG membrane emulsification and EICA with different membrane pore diameter (d_{pore} = 0.3, 0.8, and 2.0 μm). a) Uniform oil-in-water emulsion droplets from SPG membrane emulsification as well as uniform SBM microparticles after solvent evaporation. b) Size-controlled prolate ellipsoidal particles with axially stacked lamellar morphology. c) Size-controlled cross-linked prolate ellipsoidal particles. d) Size-controlled Janus discs after redispersion the corresponding cross-linked prolate ellipsoidal particles.

Characterization of the used SBM triblock terpolymer

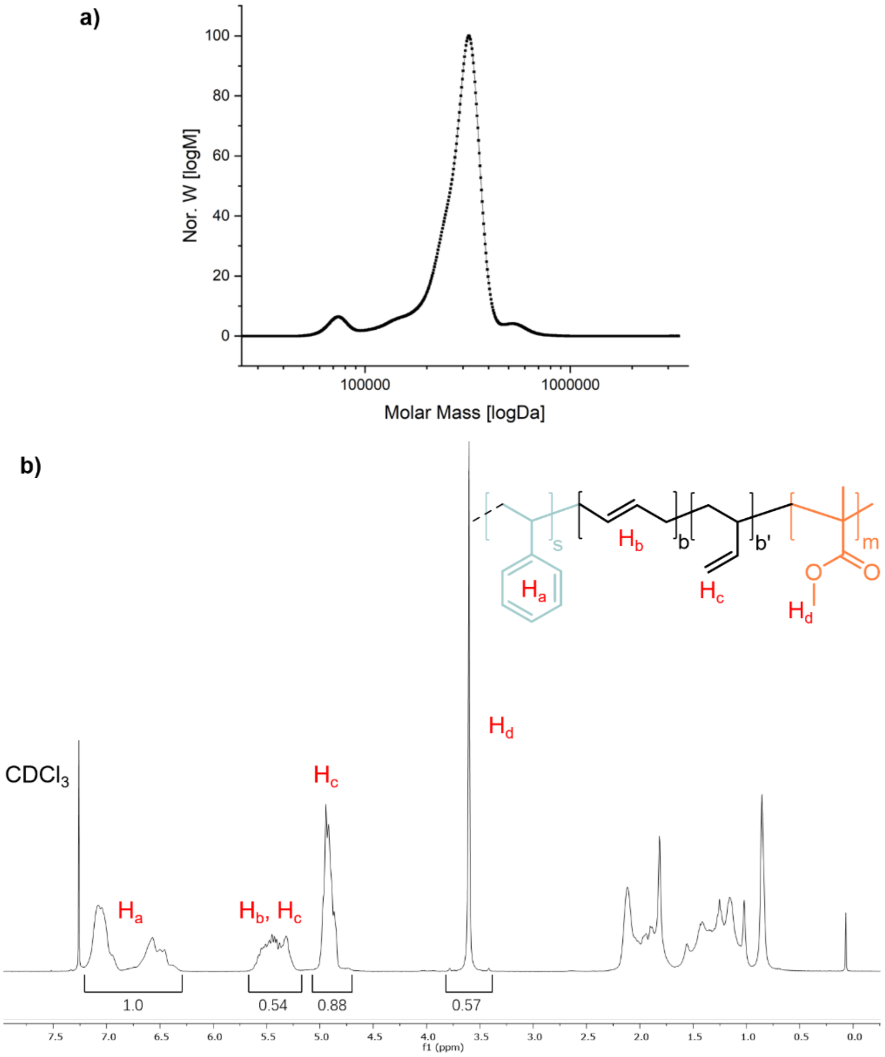

Figure S1. a) GPC and **b)** ^{1}H NMR measurement of SBM triblock terpolymer.

Grey scale analysis of SBM bulk film

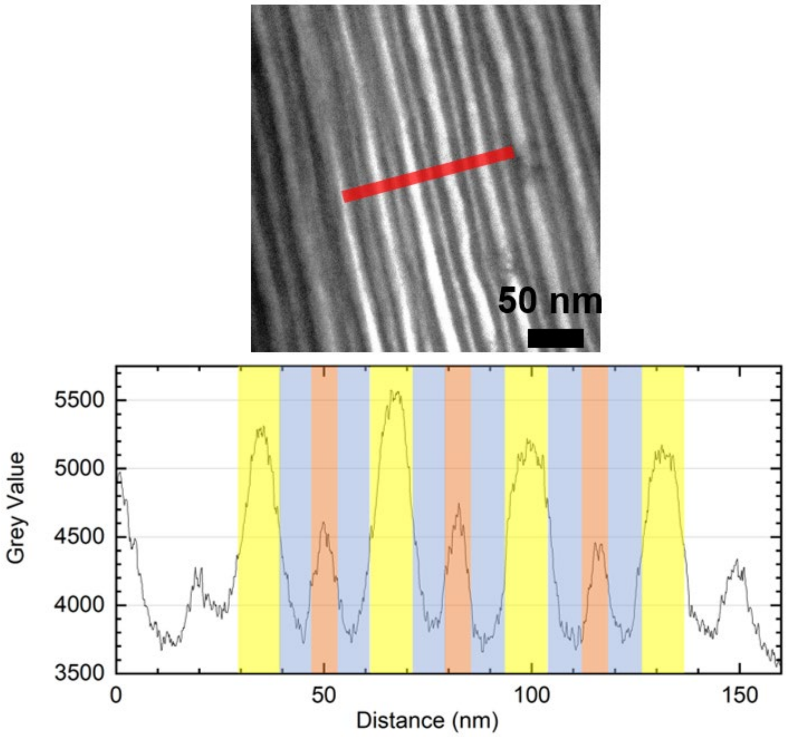

Figure S2. Upper line: TEM image of SBM bulk film. PB appears dark from OsO_4 staining and PS grey in TEM image. **Lower line:** Grey scale analysis of selected location in the upper line. Yellow, blue and orange present PS, PB, and PMMA, respectively.

Grey scale analysis of SBM prolate ellipsoidal particles

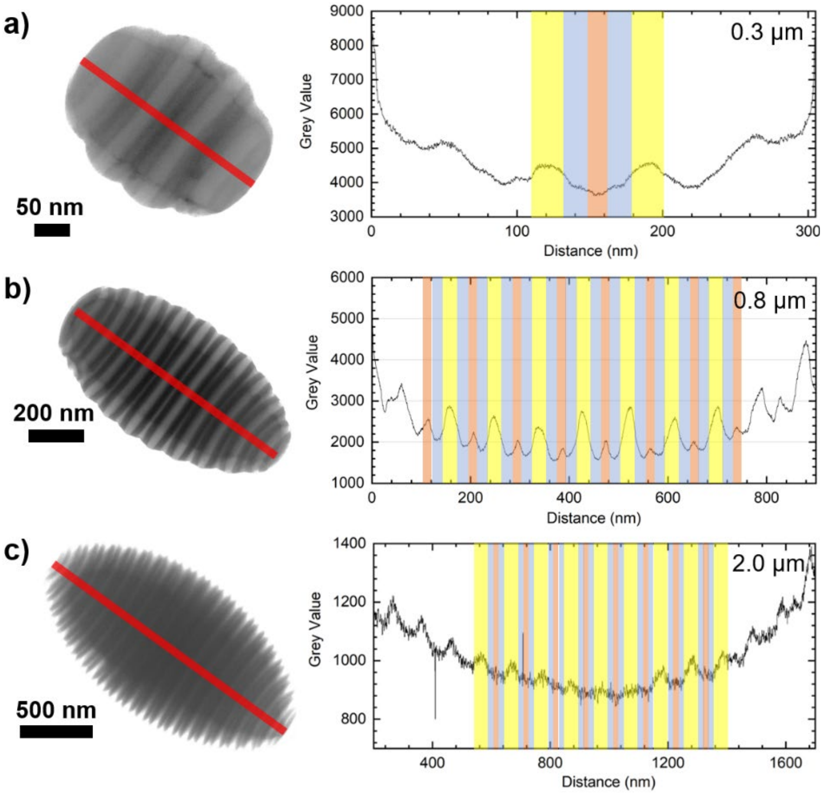

Figure S3. Grey scale analysis of selected locations of SBM prolate ellipsoidal particles with different membrane pore diameter. d_{pore} = **a)** 0.3 µm. **b)** 0.8 µm. **c)** 2.0 µm. Yellow, blue and orange present PS, PB and PMMA, respectively. PB appears dark from OsO_4 staining and PS grey in TEM images.

TEM images of Janus discs from different membrane pore diameter

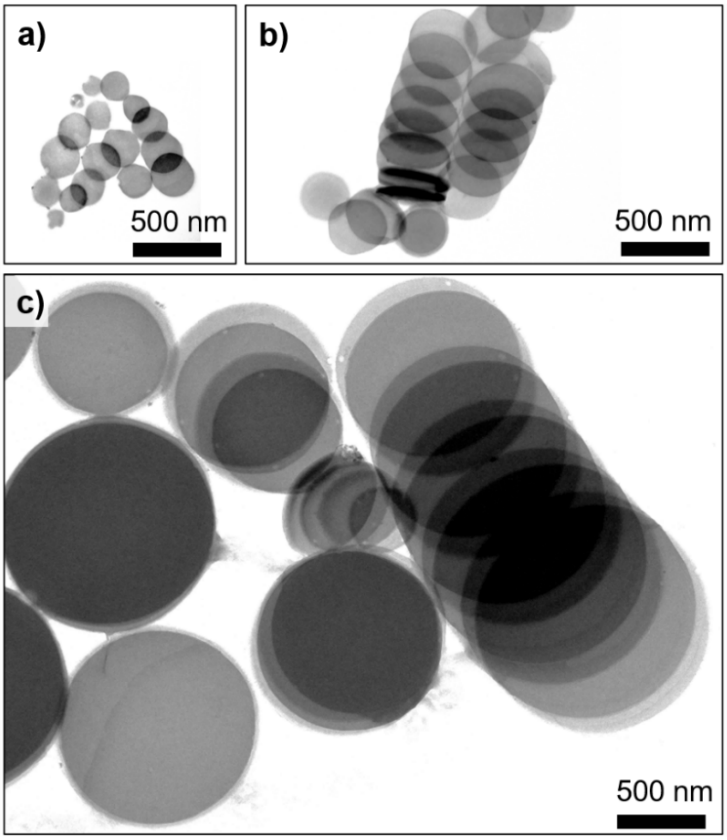

Figure S4. TEM images of Janus discs from different membrane pore diameter. a) d_{pore} = 0.3 µm, **b)** d_{pore} = 0.8 µm, and **c)** d_{pore} = 2.0 µm.

IR spectra of Janus discs before and after sulfonation

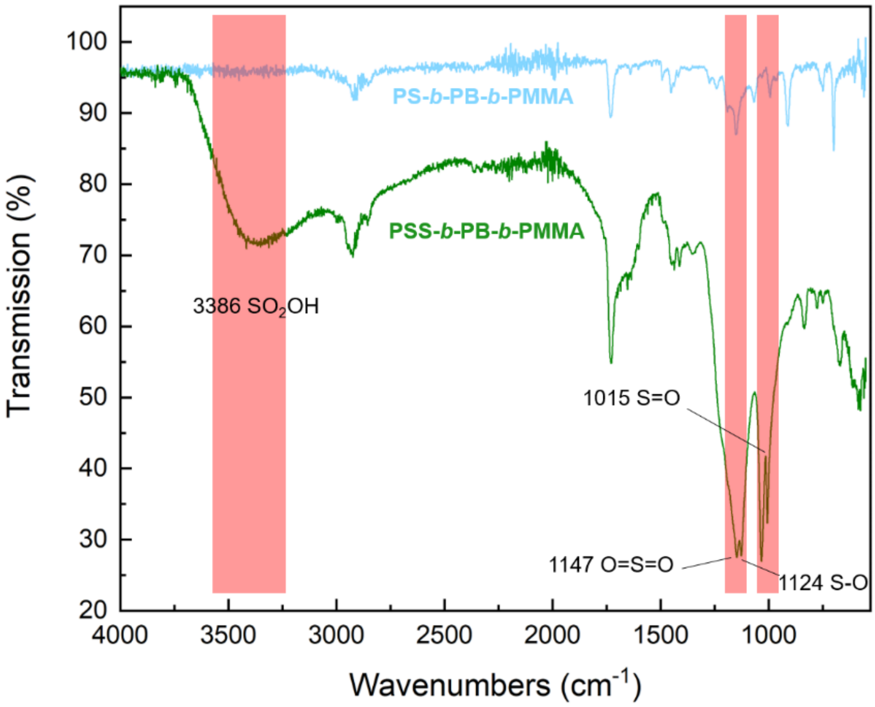

Figure S5. Blue: S_2Cl_2 cross-linked PS-*b*-PB-*b*-PMMA Janus discs from d_{pore} = 2.0 µm. **Green:** Sulfonated PSS-*b*-PB-*b*-PMMA Janus discs from d_{pore} = 2.0 µm.

TEM images of AuNPs

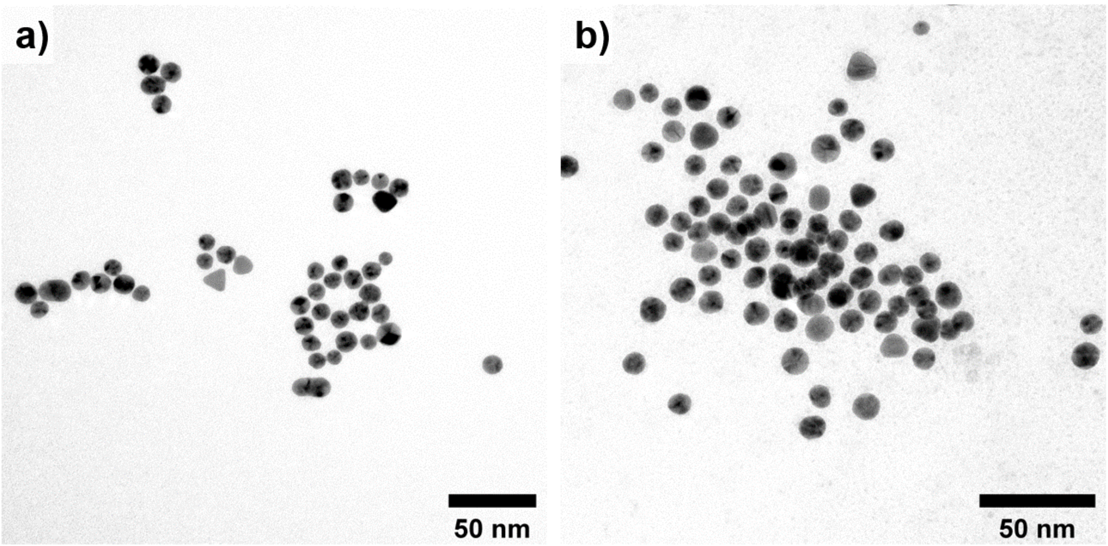

Figure S6. a) Negatively charged AuNPs. **b)** Positively charged AuNPs with an average diameter of 9-10 nm and zeta potential of +90 mV.

TEM image of sulfonated Janus discs loaded with AuNPs

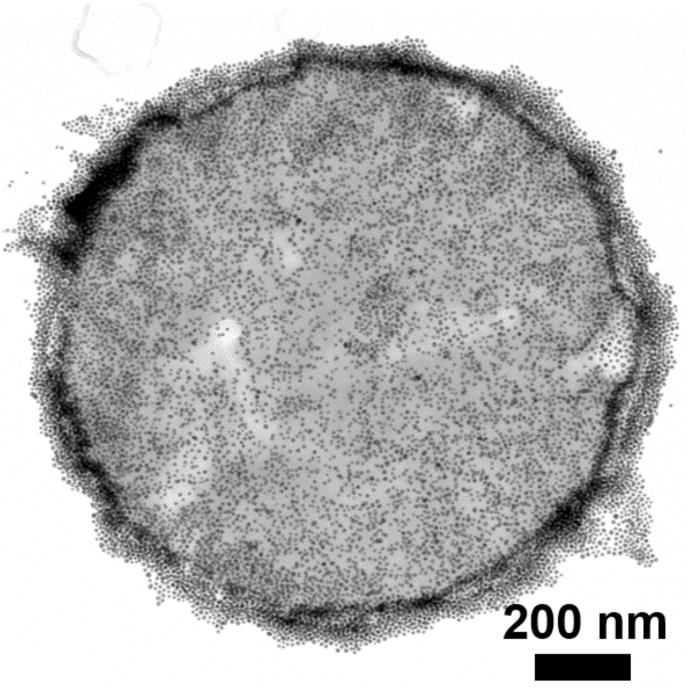

Figure S7. TEM image sulfonated Janus discs from d_{pore} = 2.0 μm loaded with AuNPs.

Emulsion stabilization of sulfonated Janus discs

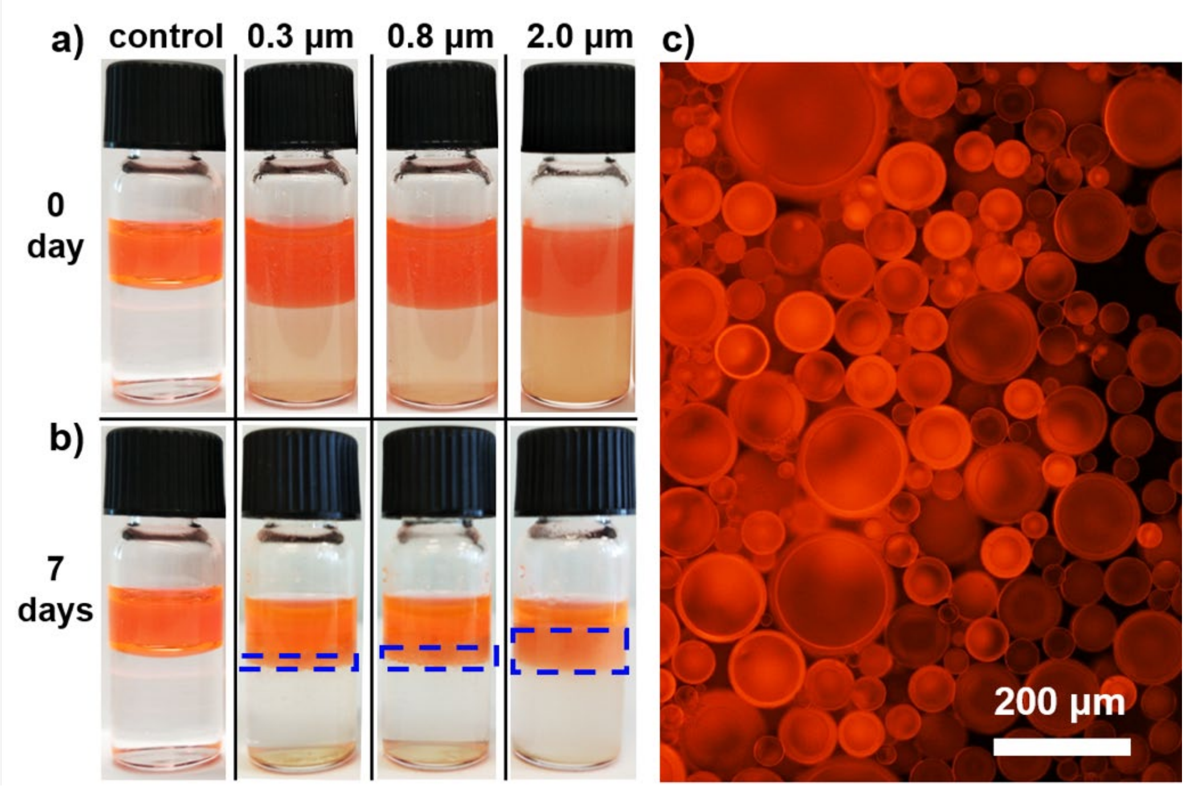

Figure S8. Emulsion stabilization of sulfonated Janus discs. Digital photographs of oil-in-water Pickering emulsions formed from toluene and water (1:2 v/v) that stabilized by sulfonated Janus discs of different size (d_{pore} = 0.3 µm, 0.8 µm, and 2.0 µm) at 0.05 wt% concentration. a) 0 day, b) 7days. The blue boxes on the lower line highlight the volume of stable emulsion. c) Fluorescence microscopy image of emulsion droplets prepared with d_{pore} = 0.3 µm sulfonated Janus discs at 0.001 wt% concentration. Toluene phase contains 0.01 wt% Nile red for contrast.

Supporting References

[1] C. Auschra, R. Stadler, *Polym. Bull.* **1993**, *30*, 257–264.
[2] J. M. Shin, M. P. Kim, H. Yang, K. H. Ku, S. G. Jang, K. H. Youm, G. R. Yi, B. J. Kim, *Chem. Mater.* **2015**, *27*, 6314–6321.
[3] T. Higuchi, A. Tajima, K. Motoyoshi, H. Yabu, M. Shimomura, *Angew. Chem. Int. Ed.* **2009**, *48*, 5125–5128.
[4] M. Müllner, T. Lunkenbein, N. Miyajima, J. Breu, A. H. E. Müller, *Small* **2012**, *8*, 2636–2640.
[5] T. Saito, B. D. Mather, P. J. Costanzo, F. L. Beyer, T. E. Long, *Macromolecules* **2008**, *41*, 3503–3512.
[6] N. G. Bastús, J. Comenge, V. Puntes, *Langmuir* **2011**, *27*, 11098–11105.
[7] J. Hassinen, V. Liljeström, M. A. Kostiainen, R. H. A. Ras, *Angew. Chem.* **2015**, *127*, 8101–8104.
[8] D. Nečas, P. Klapetek, *Cent. Eur. J. Phys.* **2012**, *10*, 181–188.
[9] S. M. Joscelyne, G. Trägårdh, *J. Memb. Sci.* **2000**, *169*, 107–117.
[10] N. Saito, Y. Kagari, M. Okubo, *Langmuir* **2006**, *22*, 9397–9402.
[11] A. J. Gijsbertsen-Abrahamse, A. Van Der Padt, R. M. Boom, *J. Memb. Sci.* **2003**, *217*, 141–150.
[12] A. J. Gijsbertsen-Abrahamse, A. Van Der Padt, R. M. Boom, *J. Memb. Sci.* **2004**, *230*, 149–159.

Author Contributions

X.Q. Data curation: Lead; Visualization: Lead; Writing—original draft: Equal; Writing—review & editing: Equal
S.F. Data curation: Supporting; Writing—review & editing: Supporting
G.Q. Data curation: Supporting; Writing—review & editing: Supporting
X.D. Data curation: Supporting; Investigation: Supporting; Writing—original draft: Equal; Writing—review & editing: Equal
C.K.W. Data curation: Supporting; Investigation: Supporting; Writing—original draft: Equal; Writing—review & editing: Equal
A.H.G. Funding acquisition: Lead; Project administration: Lead; Supervision: Lead; Visualization: Equal; Writing—original draft: Equal; Writing—review & editing: Equal.
